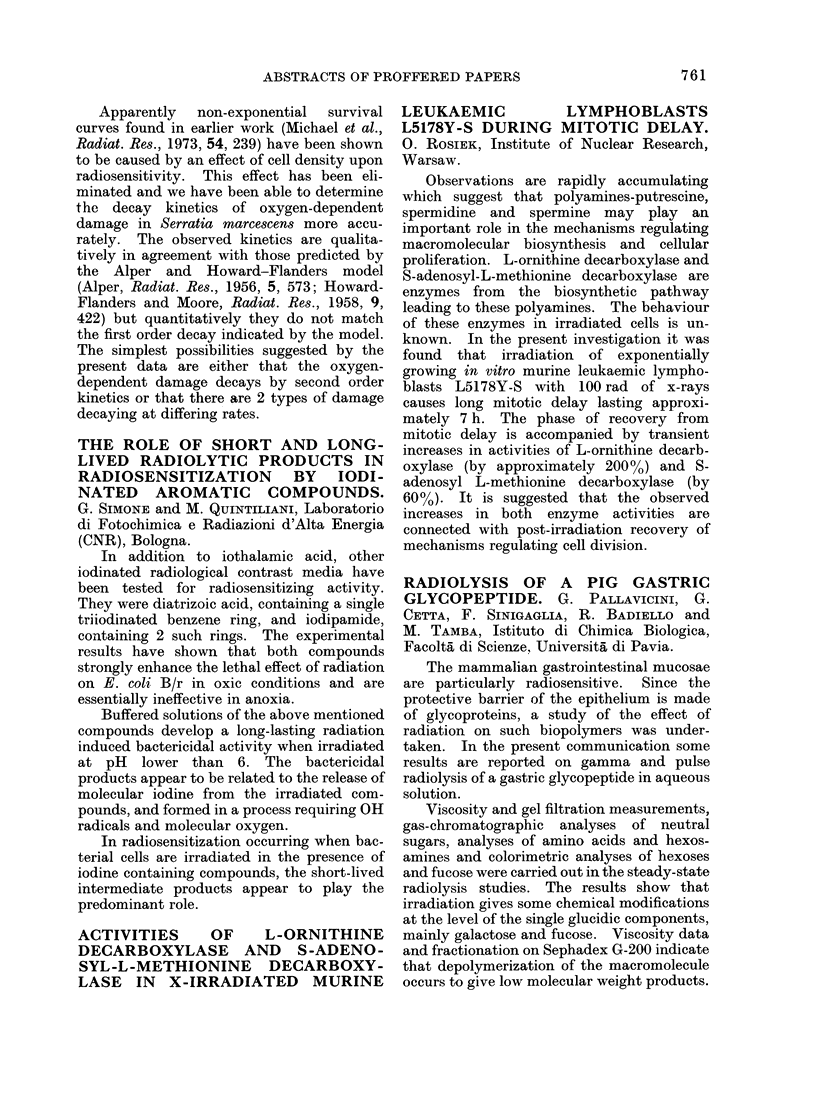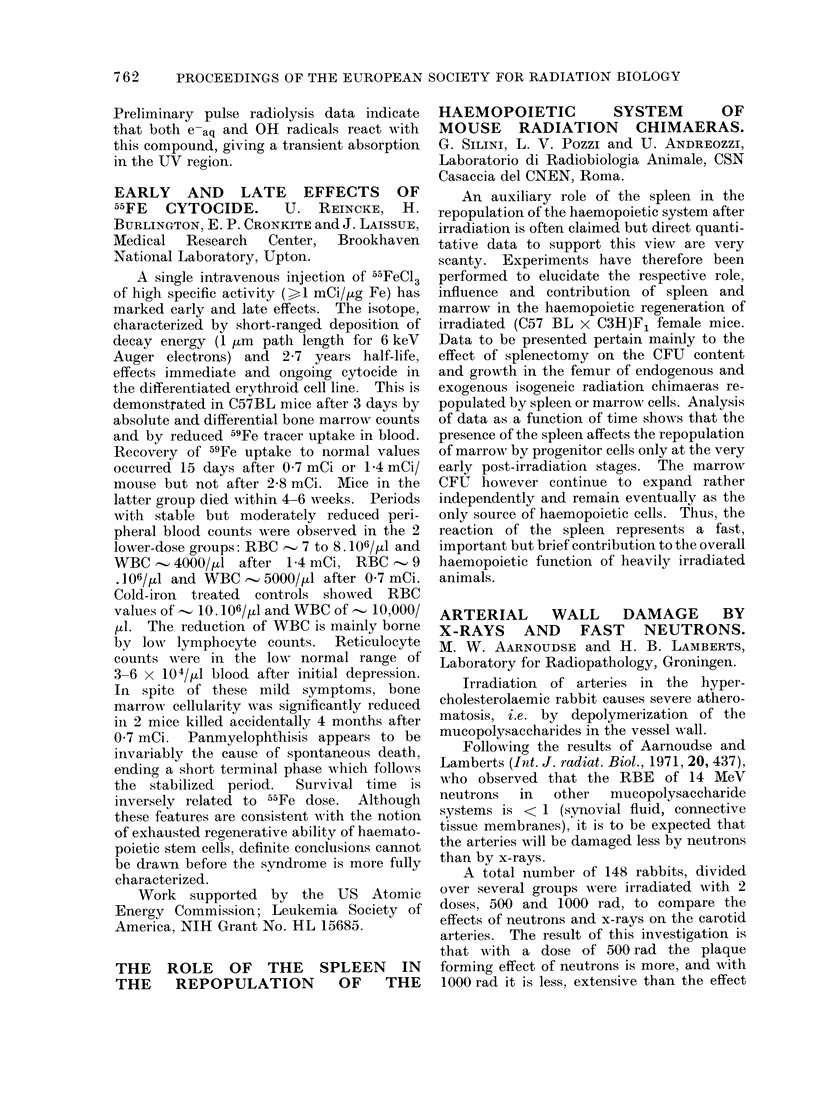# Proceedings: Radiolysis of a pig gastric glycopeptide.

**DOI:** 10.1038/bjc.1975.324

**Published:** 1975-12

**Authors:** G. Pallavicini, G. Cetta, F. Sinigaglia, R. Badiello, M. Tamba


**RADIOLYSIS OF A PIG GASTRIC GLYCOPEPTIDE.** G. Pallavicini, G. Cetta, F. Sinigaglia, R. Badiello and M. Tamba, Istituto di Chimica Biologica, Facoltā di Scienze, Universitā di Pavia.

The mammalian gastrointestinal mucosae are particularly radiosensitive. Since the protective barrier of the epithelium is made of glycoproteins, a study of the effect of radiation on such biopolymers was undertaken. In the present communication some results are reported on gamma and pulse radiolysis of a gastric glycopeptide in aqueous solution.

Viscosity and gel filtration measurements, gas-chromatographic analyses of neutral sugars, analyses of amino acids and hexosamines and colorimetric analyses of hexoses and fucose were carried out in the steady-state radiolysis studies. The results show that irradiation gives some chemical modifications at the level of the single glucidic components, mainly galactose and fucose. Viscosity data and fractionation on Sephadex G-200 indicate that depolymerization of the macromolecule occurs to give low molecular weight products.

Preliminary pulse radiolysis data indicate that both $e^-_{aq}$ and OH radicals react with this compound, giving a transient absorption in the UV region.